# How generative AI reconfigure clinician–AI and clinician–patient relationships

**DOI:** 10.1002/ctm2.70606

**Published:** 2026-02-02

**Authors:** Tianyi Shen, Xinru Wang, Yajuan Zhang, Yi Zhang

**Affiliations:** ^1^ Vanke School of Public Health Tsinghua University Beijing China; ^2^ School of Public Policy and Management Tsinghua University Beijing China; ^3^ School of Pharmaceutical Sciences Tsinghua University Beijing China; ^4^ Key Laboratory of Innovative Drug Research and Evaluation National Medical Products Administration Beijing China

The rapid uptake of generative AI (GAI) systems in clinical settings—particularly large language models (LLMs) such as DeepSeek—marks a transformative moment for healthcare and necessitates timely regulatory responses to protect safety and equity. In current practice, LLM‐based GAI is entering clinical practice through a distinctive ‘dual‐interface’ configuration. On one side, models locally deployed or fine‐tuned by healthcare institutions are embedded into hospital information systems and patient‐facing portals to streamline care processes, support clinical decision‐making and facilitate personal health management.^1^ On the other, general‐purpose models developed by companies, such as OpenAI, Google and DeepSeek, are reaching clinicians and patients through a growing ecosystem of chatbot applications.^2^, ^3^

These channels make GAI increasingly accessible to both sides of the medical encounter. Clinicians use GAI to retrieve medical knowledge, generate analytic reasoning and obtain suggestions for diagnostic and therapeutic decisions (DTD).^4^ Simultaneously, patients consult these models about symptoms, use them to interpret complex clinical rationales and review clinicians’ recommendations.^5^ This dual‐interface setting is shifting healthcare away from two separate dyads—‘clinician–patient’ and ‘clinician–AI tool’ (Figure 1A)—toward a configuration where multiple human–AI relations coexist (Figure 1A–D).

**FIGURE 1:**
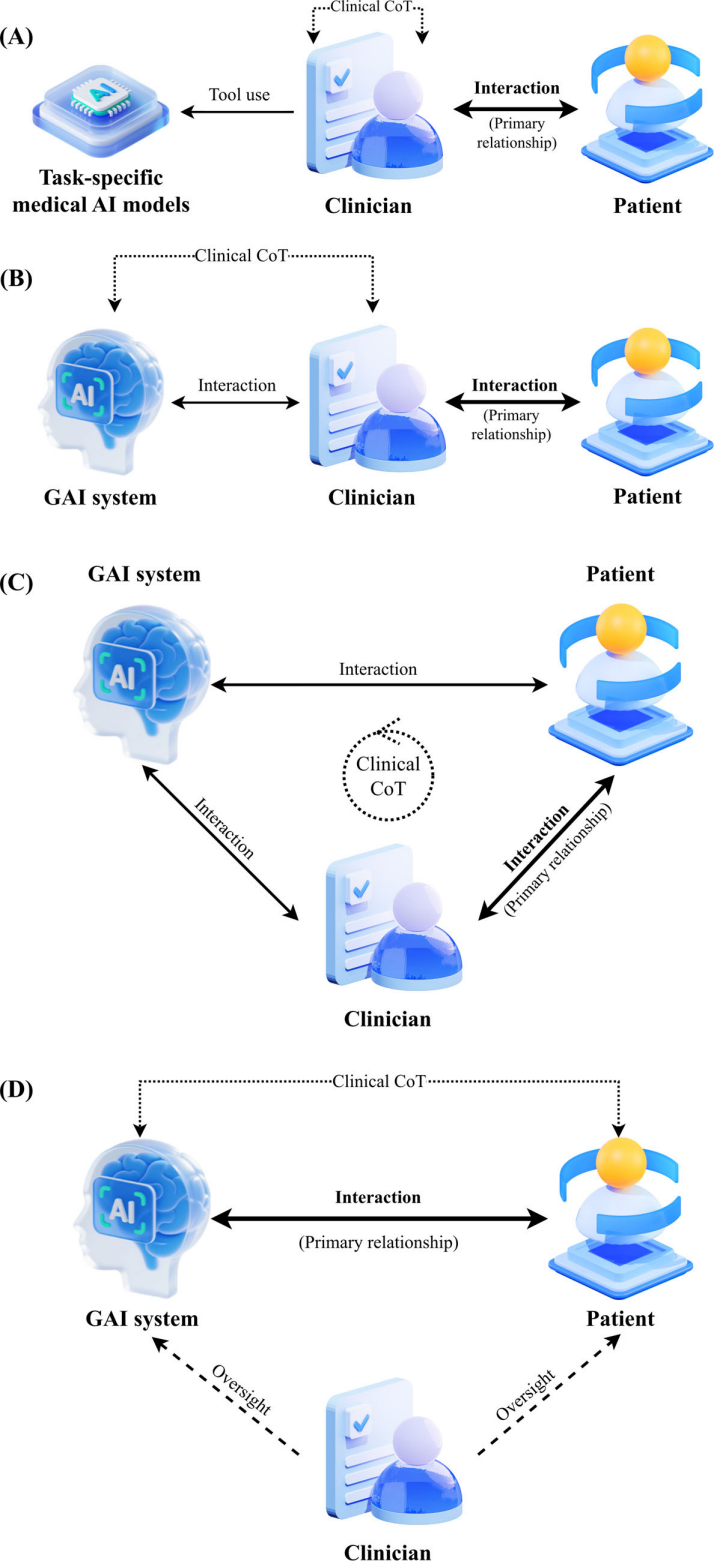
Evolving configurations of clinician–Al–patient relationships and clinical CoT participation in the generative Al era. (A) The primary relationship is the interaction between clinician and patient; the clinical CoT remains confined to the clinician, with neither AI nor patient directly involved, and task‐specific medical AI models are used by the clinician as tools. (B) The primary relationship remains the interaction between clinician and patient; the GAI system begins to function as a cognitive collaborator, interacting with the clinician and starting to participate in the clinical CoT shared between clinician and AI. (C) The primary relationship remains the interaction between clinician and patient; the GAI system begins to interact directly with the patient, forming a three‐way configuration in which the clinical CoT is shared among clinician, Al and patient, and patients can draw on AI's participation in the CoT to understand and engage with reasoning about their own care. (D) The primary relationship shifts to the interaction between the GAI system and the patient; clinicians intervene directly with patients only in specific scenarios and do not interact continuously with AI, whereas any duties to oversee or correct the clinical CoT along the Al–patient axis remain to be defined. CoT, chain‐of‐thought; GAI, generative artificial intelligence.

This Letter examines two linked axes of change. First, when GAI operates as an interactive cognitive collaborator, it enters the clinician‐led chain‐of‐thought (CoT). In configuration (B), a previously clear ‘clinician–tool’ relation becomes a clinician–AI collaboration requiring a redefinition of roles. Second, when systems capable of generating clinical CoT interact directly with patients and act as decision‐making agents, configurations (C)–(D) recast the clinician–patient relationship—its modes of explanation, patient involvement and trust—into a tripartite arrangement that explicitly includes AI.

## 1 FROM TOOLS TO COGNITIVE COLLABORATORS: CLINICIAN–AI

For decades, medical AI—from rule‐based expert systems to deep learning models—was positioned as a ‘tool’. These systems rarely prompted fundamental debate about role sharing,^6^ because they did not enter the core CoT that structures clinical practice: the process from problem formulation and evidence integration to DTD, which we refer to as the CoT for clinical reasoning and decision‐making (clinical CoT).

In the traditional configuration (Figure 1A), the clinical CoT is held exclusively by the clinician. AI systems are invoked only at specific points at the clinician's discretion. Even when they ‘compute faster’, they primarily extend perceptual capacities and function as citable evidence or auxiliary signals rather than genuine ‘speakers’ in clinical deliberation. This configuration persisted because clinical reasoning is fundamentally language‐mediated—an unfolding professional exchange where language is both the medium of intellectual interaction and the vehicle for judgment.^7^ For earlier AI, natural language understanding and generation remained a persistent bottleneck.

The advent and scaling of transformer‐based conversational LLMs such as ChatGPT have eroded this bottleneck, enabling GAI to operate in a human‐like, interactive linguistic space.^4^ GAI systems can now process medical information, integrate heterogeneous data and produce evidence‐structured reasoning along with DTD recommendations that amount to a more complete clinical CoT.^8^ In this setting, AI outputs are no longer ‘signals’ requiring human translation; they now appear as intelligible contributions directly comparable with human judgment. Consequently, GAI has shifted medical AI from a bounded tool toward a cognitive collaborator that substantively participates in the clinical CoT (Figure 1B).

This shift also generates new normative demands. Across different settings, it is now necessary to specify more precisely the legitimacy of AI involvement in the clinical CoT—for example, whether it should be confined to serving as a tool for prompting, a collaborative agent co‐generating reasoning or even an authorized decision‐maker. Correspondingly, questions arise regarding how far clinicians’ duties extend in prompting, reviewing and correcting model outputs, and where to draw responsibility boundaries for system providers regarding model design, updating and failure—all of which will require explicit responses in future regulatory frameworks.

## 2 FROM LIMITED TRANSPARENCY TO SHARED EXPLANATORY AGENCY: CLINICIAN–PATIENT

In the traditional configuration (Figure 1A,B), patients have a direct relationship only with clinicians, whereas AI remains invisible. The reasoning behind DTD largely remains within professional discourse, leaving patients with limited insight into how these decisions are made while they shoulder the outcomes and uncertainty.^9^ Meanwhile, constrained resources and heavy workloads further compress the time clinicians can devote to explanation and shared decision‐making, leaving patients’ expectations for a participatory process unmet. Such a structurally constrained setting has long been associated with disputes and breakdowns of trust in clinical encounters and makes it difficult to realize the ethical ideal of patient‐centred care in practice.^10^

In this context, advances in GAI have enabled a growing range of chatbot applications to interact directly with patients (Figure 1C,D). Patients may encounter such systems within clinical settings, where they are used to supplement clinicians’ explanations and to elaborate on different DTDs in more accessible terms, or they may access them independently before or after consultations to ask questions about their symptoms, test results and proposed treatment plans. In these ways, GAI functions as an additional, structured explanatory resource, creating a parallel channel alongside traditional clinician–patient interaction and enabling patients to engage more actively in understanding and participating in the clinical CoT that shapes their own care.

However, this parallel channel also generates institutional challenges. Although GAI may enhance transparency, divergent explanations from clinicians and GAI can create discord. If the system's reasons diverge from clinicians’ judgments, it may weaken patients’ trust. In cases of adverse outcomes, the interplay between clinician‐ and AI‐based accounts further complicates the attribution of responsibility. Moreover, if clinicians are expected to verify patient‐directed outputs or mediate three‐way exchanges, GAI can shift from sharing the explanatory workload to adding new communicative and liability burdens for clinicians. The scope of clinicians’ duties to review, qualify or override such outputs remains largely unsettled at the normative level.

In conclusion, GAI marks a structural shift from AI as a peripheral tool to a cognitive participant in the relational architecture of clinical care. In this emerging landscape, governance must prioritize layered regulatory frameworks tailored to specific clinician–AI–patient configurations, clarifying how reasoning, explanation and decision‐making authority—and their attendant liabilities—are allocated between human and artificial agents.

## AUTHOR CONTRIBUTIONS

Tianyi Shen conceived the manuscript. Tianyi Shen and Xinru Wang conducted background research and prepared the initial draft. Yi Zhang provided overall guidance and revised the manuscript. Yajuan Zhang provided expert input.

## CONFLICT OF INTEREST STATEMENT

The authors declare no conflicts of interest.

## FUNDING INFORMATION

This research was supported by the “Global Open Research Program on Sustainable Social Value (SSV Open Program)”, and the program's funding was provided by the Institute for Sustainable Social Value, Tsinghua University.

## ETHICS STATEMENT

The authors have nothing to report.

## CONSENT

The authors have nothing to report.

## Data Availability

Data sharing not applicable to this article as no datasets were generated or analysed during the current study.
